# Dilemma of Supra- or Infrapatellar Tibial Nailing: Anterior Knee Pain vs. Intra-Articular Damage

**DOI:** 10.1155/2022/8220030

**Published:** 2022-04-21

**Authors:** Levent Umur, Enes Sari, Serdar Orhan, Serkan Sürücü, Cengiz Yildirim

**Affiliations:** ^1^Acıbadem Kadıköy Hospital, Orthopedics and Traumatology Department, Tekin Sokak No. 8 Acıbadem Kadıköy, Istanbul, Turkey; ^2^Orthopaedics and Traumatology Department, Near East University Hospital, Mersin-10, Turkey; ^3^Orthopaedics and Traumatology Department, Sultan Abdulhamid Han Training and Research Hospital, Uskudar, Istanbul, Turkey; ^4^University of Missouri, Kansas, Department of Orthopedic Surgery, USA

## Abstract

**Aim:**

Intramedullary nailing (IMN) is widely accepted as the treatment of choice for tibial fractures, and a suprapatellar method has been described to prevent common problems associated with the typical infrapatellar IMN technique, such as anterior knee pain. However, in the suprapatellar technique, injury to intra-articular structures is a concern. The aim of this study was to compare the clinical and radiological results of suprapatellar and infrapatellar IMN in terms of union, complications, and function.

**Methods:**

A retrospective evaluation of 61 patients who had undergone suprapatellar (*n* = 29, Group A) or infrapatellar (*n* = 31, Group B) tibial IMN was conducted. For the suprapatellar group, magnetic resonance imaging scans were acquired on the sixth month follow-up. Complications, radiological findings, functional outcomes, surgery duration, and differences in a range of motion (ROM) were compared.

**Results:**

Surgery duration was significantly shorter in Group A (81 mins vs. 107 mins, *p* < 0.001), and visual analog scale (VAS) values were significantly higher in Group B (0.17 vs. 1.62, *p* < 0.001). In Group A, the patients' Lysholm scores were significantly higher (95.6 vs. 92, *p*=0.006). In terms of anterior knee pain, none was experienced in Group A (0%), while 11 patients (26.1%) reported about it in Group B. There were no statistically significant differences between the two groups in SF-36 score (*p*=0.925), the radiographic union scale in tibial (RUST) fractures score (*p*=0.454), union time (*p*=0.110), or ROM (*p*=0.691). In Group A, two cases of patellofemoral cartilage degeneration were observed.

**Conclusion:**

If performed with sufficient expertise, the suprapatellar IMN technique is a safe, reliable technique with a low frequency of anterior knee pain for treating tibial fractures. There is no clear evidence that it causes damage to intra-articular structures. The possibility of patellofemoral cartilage degeneration due to this technique should be further evaluated by prospective studies including pre- and postoperative radiologic assessments.

## 1. Introduction

Tibial fractures are the most common long bone fractures, occurring most commonly as a result of torsional injury, motor vehicle accident, or gunshot wound, respectively [1]. The most common problems associated with treating such fractures are the anteromedial tibia's inadequate soft tissue coverage, which results in a higher rate of open fracture than other long bones. Additionally, the tibial diaphysis has low vascularity. All of these variables contribute to the difficulty of treating tibial fractures and might result in delayed healing and increased consequences.

The treatment of a tibial fracture sought to restore the extremity's correct alignment, allowing the patient to achieve an acceptable level of function and to become mobile as rapidly as feasible. Although treatment strategies for the tibial shaft and extra-articular tibia proximal fractures have evolved in favor of intramedullary nailing, open reduction and internal fixation techniques remain the gold standard for tibial plateau and tibia proximal fractures with intra-articular extension, as articular reduction is critical for improving functional and clinical outcomes [2]. Tibial intramedullary nailing (IMN) is regarded as the golden standard approach for treating these diaphyseal fractures because it provides excellent fracture stability, eliminates malalignment, allows for early motion and weightbearing, and lowers the occurrence of complications [3]. While the infra-patellar approach is the traditional IMN technique for tibial fractures, it presents several difficulties, including difficulty in obtaining optimal intra-operative fluoroscopic images, sustaining anatomical reduction due to hyperflexion, aligning of valgus and procurvatum, and swelling of the extremity, which increase the risk of compartment syndrome and lengthen the surgical duration [4]. Additionally, anterior knee pain was the most often reported complication of the infrapatellar method, occurring in 10%–86% of patients [5].

A suprapatellar approach has been described to overcome these obstacles and prevent the complications associated with them. This technique has gained popularity because it facilitates initial and subsequent fracture reduction maintenance, enhances anteroposterior and lateral fluoroscopic visualization, aids in more precise nail implantation, reduces operation time, and decreases the risk of nonunion [6]. However, a significant concern with the suprapatellar technique is an increased risk of damaging intra-articular structures, especially the patellofemoral cartilage [7, 8].

The purpose of this study was to compare the clinical and radiological outcomes of two IMN techniques in terms of union rate, complication rate, and functional outcome. The critical question was which factor was a greater risk factor for tibial IMN: anterior knee pain or iatrogenic intra-articular lesions.

## 2. Materials and Methods

### 2.1. Study Patients

**Figure 1:**
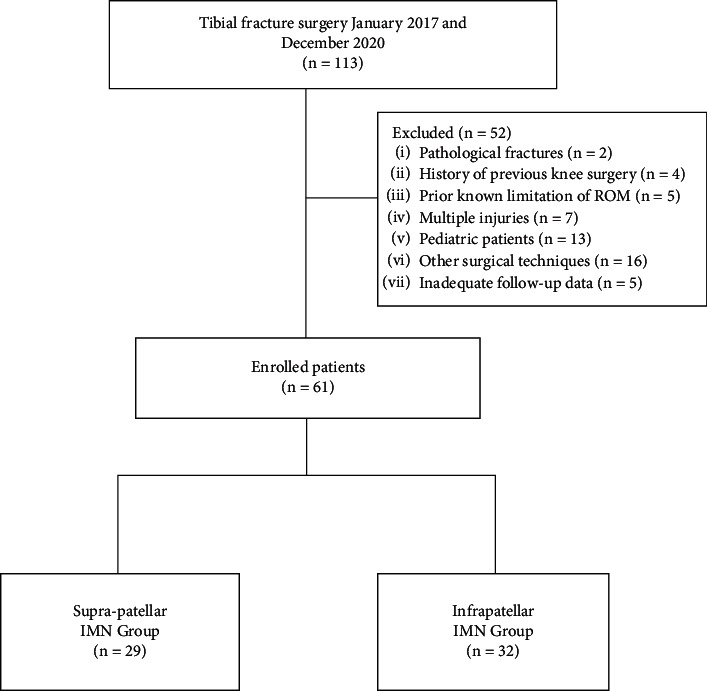
Study flow chart.

Between January 2017 and December 2020, patients who underwent tibial fracture surgery were assessed retrospectively. This retrospective cohort research included patients with tibial fractures without an intra-articular extension who were treated with IMN. Patients with pathological fractures, a history of previous knee surgery, a previously documented restriction of range of motion, multiple injuries, pediatric patients, patients treated with a technique other than IMN, and patients with insufficient follow-up data were excluded (Figure 1). All cases in each group were operated by the same surgical teams within themselves. There was no need for randomization or any reason to cause a selection bias because we have two separate equally experienced surgical teams using only one technique or the other. Therefore, we included all patients meeting inclusion and exclusion criteria for each group since all patients were randomized automatically at the admission depending on which team is on call.

### 2.2. Surgical Technique

**Figure 2:**
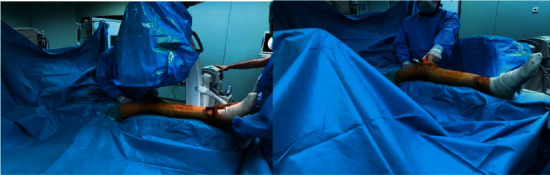
Positioning of the patient with a right-trapezoid padding and fluoroscopic imaging of all lower extremity without changing the extremity position in both AP and lateral views in the suprapatellar approach.

**Figure 3:**
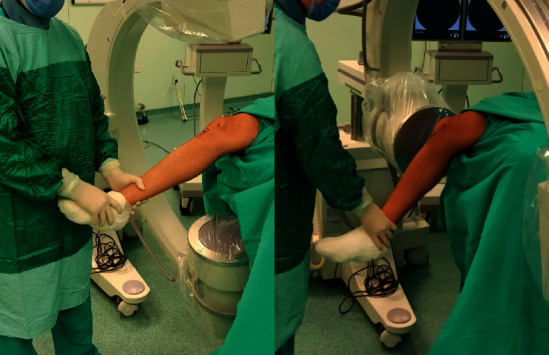
Positioning of the patient and use of fluoroscopy for AP and lateral views in the infrapatellar approach.

Both techniques incorporated the Smith & Nephew Trigen Meta-Nail, as well as a semi-extended instrument set for the suprapatellar technique. The suprapatellar technique was utilized through a 3 cm suprapatellar longitudinal incision without medial extension to avoid injury to the retinaculum and patellofemoral ligaments, while the affected limb was placed on a right-trapezoid foam pad (Figure 2) with the knee at 30–40° of flexion. The infrapatellar approach was carried out through a medial parapatellar incision as the affected limb was hanging off the edge of the operating table and the knee was in 90° of flexion (Figure 3). Proximal locking screws were applied through the guide and distal locking screws were applied with a magnetic distal targeting system for both groups. During surgery, for all suprapatellar cases, the knee joint was irrigated with 1500 cc saline solution after the nailing procedure was completed. Patients are typically allowed and encouraged to begin full weight-bearing (FWB) immediately following surgery, except segmental and extremely comminuted (AO Type 42-B3, 42-C2, and 42-C3) patients. These patients are allowed for partial weight-bearing (PWB) as tolerated for three weeks.

### 2.3. Data Collection

Our institution routinely performs MRI evaluations on all suprapatellar tibial IMN patients at six months to rule out iatrogenic damage to intra-articular structures. We used a 6-month time frame to rule out any early postoperative alterations or new diseases that may arise after regaining full daily functioning. Following discharge, follow-up examinations were scheduled for the first week, the third week, the sixth week, the third month, and every month thereafter, until complete union and function were obtained without clinical complaints. Six-month follow-up functional and radiological assessments, as well as clinical outcome scores, were used. Patient demographics, fracture type, associated injuries, operation duration, type of anesthesia, full weight-bearing time, follow-up duration, visual analog scale (VAS), anterior knee pain, the Lysholm score, SF-36 score, radiographic union scale in tibial (RUST) fractures score, time of union, the difference in range of motion (ROM) with the uninjured side, and MRI findings of cartilage degeneration according to the modified Outerbridge grading scale were all evaluated.

### 2.4. Statistical Analysis

Descriptive data were used to define the variables, and the Mann–Whitney *U* test was used to compare the variables between the groups since the data were not normally distributed according to the Shapiro–Wilk test. Statistical significance was set at *p* < 0.05. The statistical analysis was performed using IBM SPSS Statistics 22.0 (IBM Corp., Armonk, N.Y., USA).

## 3. Results

There were a total of 113 tibial fractures, and 61 met the inclusion criteria, including 29 and 32 cases in the suprapatellar (A) and infrapatellar (B) groups, respectively. The mean age was 35.6 ± 11.7 years (range, 18–64 years) for Group A and 33.09 ± 8.4 years (range, 21–59 years) for Group B, respectively. The mean period of follow-up for all patients was 8.7 months (range, 6–27 months) (mean ± SD; Group A 8.5 ± 3.42 months, Group B 7.8 ± 4.94). Seven patients in Group A (*n* = 29) and eight patients in Group B (*n* = 32) had open fractures. According to the AO classification, there was no significant difference in dispersion between groups (Table 1). The mean surgical time was 81 ± 10.21 minutes (range, 60–100 mins) for Group A and 107 ± 15.49 minutes (range, 90–150 mins) for Group B, making it significantly longer in Group B (*p* < 0.001). For VAS values, the mean was 0.17 ± 0.65 (range, 0–3) for Group A and significantly higher (*p* < 0.001) at 1.62 ± 0.94 (range, 0–3) for Group B. The Lysholm scores for Group A were significantly higher (*p*=0.006) than those for Group B at a mean of 95.6 ± 5.38 (range, 83–100) and 92.03 ± 6.14 (range, 70–100), respectively. There were no statistically significant differences in the SF-36 score (*p*=0.925), RUST score (*p*=0.454), union time (*p*=0.11), or ROM (*p*=0.691) between the groups. There were three patients in Group A and four patients in Group B who were not allowed FWB for three weeks. Demographic and statistical data are presented in Table 2. In two of the 29 patients who were evaluated with MRI, cartilage degeneration was seen after six weeks, but no damage to other intra-articular structures was experienced. No patients reported anterior knee pain in Group A (0%), with 11 experiencing it in Group B (26.1%).

## 4. Discussion

The most important finding of this study is that the suprapatellar technique prevents anterior knee pain after tibial IMN even in the early postoperative period. The results of our study also showed that Lysholm scores are significantly higher and surgery durations are significantly shorter for the suprapatellar group, compared to the infrapatellar approach.

### 4.1. Anterior Knee Pain vs. Intra-Articular Damage

Anterior knee pain and alignment issues are the most common complications of tibial IMN. Seven patients underwent suprapatellar IMN without the use of a protective sleeve in a study conducted by Jakma et al. Following that, an arthroscopic assessment of the patellar and trochlear cartilage was performed, and no patient reported anterior knee pain despite the presence of cartilage damage [9]. Gelbke et al. assessed the pressure on the patellofemoral joint during IMN and found a peak contact pressure of 3.83 MPa in a suprapatellar treatment group, which is significantly higher than the maximum of 1.26 MPa observed in infrapatellar procedures [10]. This seems controversial, but it has also been demonstrated that chondrocyte damage does not occur until 4.5 MPa, that this is dose-related, and that patellofemoral pressure can increase up to 4–5 MPa by flexion at 120–135° [11, 12]. These findings suggested that prolonged knee hyperflexion has a negative effect on patellofemoral cartilage vitality.

The findings of this study are in line with these conclusions. Only two of the 29 suprapatellar patients evaluated by MRI demonstrated radiographic evidence of cartilage degeneration using the modified Outerbridge grading scale for chondromalacia but still no clinical complaints. The two patients had Grade 1 and Grade 2 degeneration following high-energy trauma of falling from height and a pedestrian-motor vehicle accident, respectively, and so it is hard to conclude if the incidents or the suprapatellar IMN caused chondral degeneration. Al-Azzawi et al. [13] demonstrated a need for this type of documentation of the patellofemoral cartilage condition following suprapatellar IMN in a recent study, and the results presented here may address that requirement. Additional research, including pre- and postoperative MRI evaluations, will provide more definitive evidence on this subject.

With a prevalence of up to 86%, post-operative anterior knee pain is the most prevalent consequence following tibial IMN [14]. Iatrogenic cartilage degeneration, osseous window damage, infrapatellar nerve damage, meniscal injury, fat pad damage, patellar tendon damage, or prolonged knee flexion during surgery can all cause this pain. The effect of nail entry point localization was evaluated in many studies. Tendon violation, morphologic changes in the tendon structure, and infrapatellar nerve damage are stated as the main reasons for anterior knee pain [5, 15]. On the other hand, numerous studies have concluded that transtendinous or paratendinous approaches have little effect on the prevalence of anterior knee pain [16–19]. According to Lovell et al. [16], splitting or lateral retraction of the patellar tendon did not affect the prevalence of anterior knee pain. Vaistö et al. [18] conducted a prospective randomized study with an eight-year follow-up and concluded that using a paratendinous technique did not result in a reduction in the prevalence of anterior knee pain. As the aim of this study was not to compare knee pain in different infrapatellar techniques, we routinely performed the infrapatellar approach via a transtendinous technique, which is the preferred method.

The suprapatellar approach is considered one of the semi-extended tibial nailing techniques. The other semi-extended techniques include lateral or medial parapatellar approaches. Although the parapatellar approach was described as an extra-articular technique, an incision through retinaculum or patellofemoral ligaments may result in patellofemoral instability or anterior knee pain since the retinaculum has high nerve fiber content [20]. Therefore, we preferred the suprapatellar approach as a semi-extended technique, and we did not utilize the medial extension of the incision.

El Moumni et al. [21] reported that anterior knee pain can be seen in up to 23% of patients following retrograde femoral nailing which, like supra-patellar IMN, is performed in a semi-extended position though it utilizes a different osseous window. As such, hyperflexion does not seem to be the only cause of anterior knee pain. In this study, no patient in Group A experienced anterior knee pain, while 11 of the 42 patients (26.1%) in Group B reported it.

Other intra-articular structures that are considered at risk in the suprapatellar approach are the inter-meniscal ligament, anterior cruciate ligament, and the anterior horns of the menisci [6]. Gaines et al. [8] reported two inter-meniscal ligament injuries in a study on 10 fresh frozen cadaver specimens. No meniscal or cruciate ligament injuries were seen in the suprapatellar group and three inter-meniscal ligaments and one medial meniscus anterior horn injury were documented in the infrapatellar group. It is stated that even if the difference was not statistically significant due to the small sample size, the suprapatellar approach is with a lower incidence of injury to intra-articular structures. No radiological signs of damage to these structures were identified by MRI, and this may be the result of the technique's easier fluoroscopic positioning, and therefore, more precise determination of entry point is obtained.

### 4.2. Reduction and Alignment

The suprapatellar approach is more likely to be used in proximal 1/3 tibial fractures. Obtaining and maintaining reduction can be difficult in infrapatellar tibial IMN, especially for proximal and distal (AO types 41–43) fractures, and this can lead to alignment problems, longer operation times, and increased exposure to radiation [21]. More specifically, increased fracture-deforming forces can occur through hyperflexion of the knee; obtaining suboptimal fluoroscopic anteroposterior and lateral images can be problematic; maintaining reduction on a hovering extremity is difficult, and the risk of compartment syndrome is increased in a hanging extremity. Recent studies have shown that the suprapatellar approach to tibial IMN can achieve an excellent alignment [1,4] and reduce operation times and radiation exposure [13, 22].

Although the purpose of this study was not to evaluate alignment and no particular measurements were taken, the results are consistent with previous findings, since neither coronal nor sagittal plane abnormalities were identified. There were no alignment problems detected in the suprapatellar group, even for proximal (AO type 41, *n* = 2) and distal (AO type 43, *n* = 3) fractures. terior ankle pain occurred as an additional consequence in three individuals with AO type 43 fractures following the use of a third distal locking screw on the anteroposterior plane. This was most likely caused by the neurovascular bundle's closeness to the screw location [23].

### 4.3. Surgery Duration

Another finding of this study was that suprapatellar technique is associated with shorter surgery durations. This may be caused by more than one reason. First, achieving and maintaining reduction is easier with this technique, especially with a trapezoidal padding instead of cylindrical in contrast to existing studies that utilize wedge or cylindrical paddings [13]. Once reduction is acquired, there was almost no reduction loss during the process. This stability is also associated with fluoroscopic imaging, which may be the second reason because this technique provides the use of preset fluoroscopy positions without repositioning the extremity. Wang et al. [22] reported in a meta-analysis of studies comparing two techniques that the suprapatellar technique is associated with less fluoroscopy time but no difference in surgery durations.

### 4.4. Follow-Up Time

There are multiple studies and meta-analyses in the literature comparing the extent of knee pain between supra and infrapatellar approaches [5, 6, 24, 25]. The common point of most of these studies was that they all concluded that there was no difference between the two approaches regarding anterior knee pain by evaluation of scores over longer periods (12 months and above). Studies with shorter follow-up times, such as the one presented here, show that the suprapatellar approach was associated with lower anterior knee pain incidence and faster recovery, even in the early postoperative period [5]. Although longer study periods were favorable for evaluating long-term results, we believed that we need studies to determine and choose the least restrictive treatment methods essential to provide patients with the shortest and safest treatment. Therefore, this study was designed to be late enough to evaluate and be free of early postoperative impairments but early enough to be free of new injuries.

### 4.5. Functional Scores

In terms of functional scores, there were significant differences in the Lysholm and VAS scores between groups in favor of the suprapatellar group, but no difference was found for SF-36 scores. We think that this difference might be caused by the specificity of the Lysholm score to the knee, unlike SF-36. Similar results were also reported in the literature about the Lysholm score being significantly better for the suprapatellar technique [26].

Additionally, the suprapatellar technique raises concerns of heterotopic ossification from reaming debris in the joint and septic arthritis, particularly in open fractures [14, 27]. To avoid these complications, knee joints were irrigated with 1500 cc saline solution following each technique; no incidences of intra-articular contamination were observed.

The limitations of this study include its retrospective methodology, the lack of preoperative MRIs, and a sample size that was insufficient to fully justify the results. Since the patients with chondral damage are high-energy trauma patients, it is possible that the damage was produced by the initial trauma, but the lack of preoperative MRIs precludes this theory. Thus, prospective randomized studies with larger groups, including pre- and postoperative MRI evaluations would provide more accurate and objective responses to current concerns about suprapatellar IMN of tibial fractures.

## 5. Conclusion

In comparison to the conventional infrapatellar procedure, suprapatellar IMN improves the outcomes by reducing anterior knee pain, enabling fracture reduction and fluoroscopy positioning, and shortening the duration of the operation. The risk of patellofemoral cartilage damage with the suprapatellar approach needs to be documented clearly by prospective studies including pre- and postoperative radiological assessments but yet there is no concrete evidence that it causes any other intra-articular lesions.

## Figures and Tables

**Table 1:** Fracture type distribution according to AO classification.

AO fracture type		Suprapatellar	Infrapatellar
41	A	2	1
42	A	17	21
	B	4	7
	C	3	3
43	A	3	0

**Table 2:** Demographics and statistical analysis of variables.

Variables	Suprapatellar (*n* = 29)	Infrapatellar (*n* = 32)	*p*-value
VAS score	0.17 ± 0.65	1.62 ± 0.94	<0.001^*∗*^
Lysholm score	95.6 ± 5.38	92.03 ± 6.14	0.006^*∗*^
SF-36 score	88 ± 10.8	85 ± 6.13	0.925
RUST score	12 ± 1.4	10 ± 1.29	0.454
Union time (weeks)	18 ± 3.55	20 ± 11.56	0.11
Surgery dur. (min.)	81 ± 10.21	107 ± 15.49	<0.001^*∗*^
ROM dif. (degree)	5 ± 3.8	5 ± 3.9	0.691

^*∗*^: *p* < 0.05, statistically significant values are marked. Values are provided as mean ± SD.

## Data Availability

The data that support the findings of this study are available from the corresponding author upon reasonable request.
